# Radiotherapy Can Cause Haemostasis in Bleeding Skin Malignancies

**DOI:** 10.1155/2012/168681

**Published:** 2012-12-30

**Authors:** Helena Sung-In Jang, Andrew Spillane, Frances Boyle, Gerald Fogarty

**Affiliations:** ^1^St Vincent's Hospital Sydney, Victoria Street, Darlinghurst, NSW 2010, Australia; ^2^Melanoma Institute of Australia, Poche Centre, Rocklands Road, North Sydney, NSW 2060, Australia; ^3^Department of Radiation Oncology, Mater Hospital, P.O. Box 1003, Crows Nest, NSW 2065, Australia

## Abstract

Radiotherapy (RT) can cause haemostasis in select cases of malignant bleeding. We present two cases where RT was used to prevent fatal exsanguination from bleeding skin malignancies. Treatment was with radical intent in one case and palliative intent in the other. The dose used in both cases was 20 Gray (Gy) in 5 fractions. To our knowledge, this is the first report of radiation-induced haemostasis in bleeding skin malignancies.

## 1. Introduction

Radiotherapy (RT) can cause haemostasis in malignant bleeding in many cancer types [1–5]. This paper describes two cases in which the effect of RT was lifesaving in haemorrhagic cutaneous skin malignancies. A radical case is described in a neoadjuvant setting for a large malignant fibrous histiocytoma (MFH) of the scalp skin. A palliative case is also presented which used radiation for melanoma metastases of the small bowel. To our knowledge, this is the first report of radiation-induced haemostasis in bleeding skin malignancies.

## 2. Case Presentation

**Case 1**

A 70-year-old man was referred in June 2011 with torrential bleeding from a large nonhealing cutaneous lesion on the scalp. The lesion had been growing for more than one year. Histopathologic examination of the biopsy specimen showed MFH. A computed tomography (CT) scan of the brain was performed and showed a 100 millimetre heterogeneous enhancing scalp mass abutting the periosteum. Staging positron emission tomography—computed tomography (PET-CT) scan found no evidence of metastatic disease. The patient was then commenced on anticoagulants for lower limb deep venous thrombosis. The scalp started to bleed when the scab was accidentally knocked off the wound. He required multiple blood transfusions. Multiple interventions to stop the bleeding had been trialled including corticosteroid injections, betadine dressing, flamazine, and cauterisation. Haemostasis was only attained by compression bandage. Attempts at bandage removal caused rebleeding.

**Figure 1:**
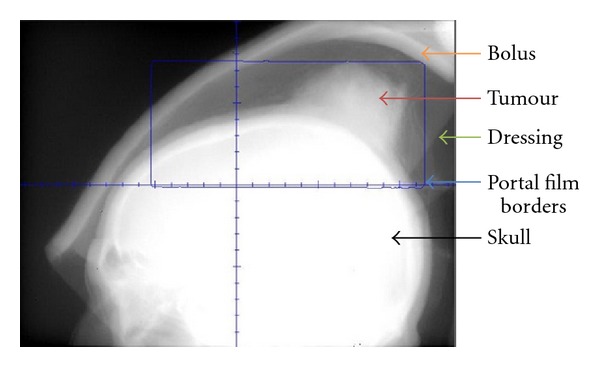
Lateral port film showing bolus, dressing, and the lesion. In this image, the beam irradiates the tumour with the dressing in place. Short course radiotherapy of 20 Gy in 5 fractions was used for the initial part of treatment.

**Figure 2:**
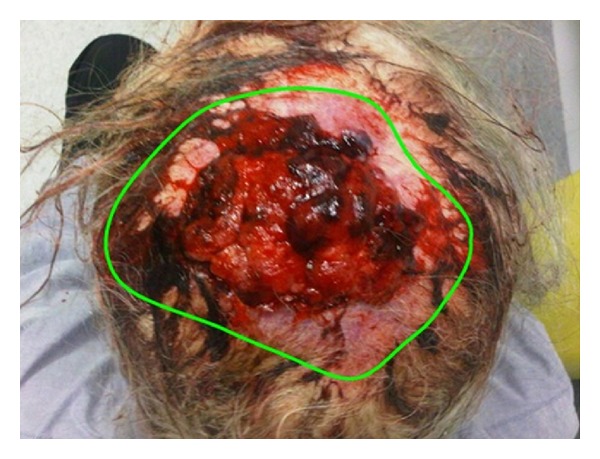
Superior view of the lesion after initial 20 Gy and postdressing removal. A fortnight after the first 20 Gy, acute haemostasis was achieved to enable safe removal of compression bandage. The green line delineates the lesion.

**Figure 3:**
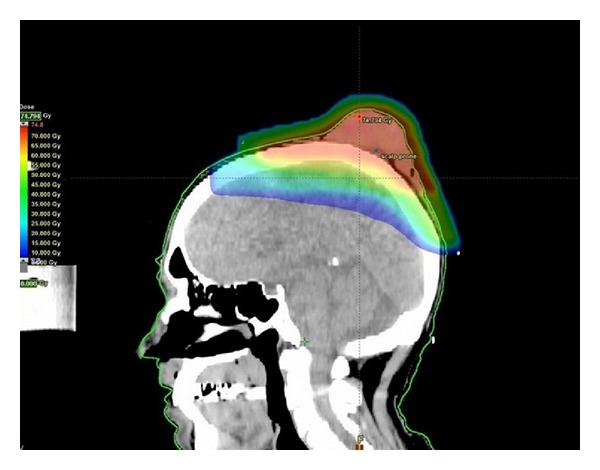
Dosimetry of VMAT plan for the patient of Case 1. After a rest period of fourteen days, further 40 Gy/20 fractions of radiotherapy were administered to allow further tumour shrinkage prior to resection.

**Figure 4:**
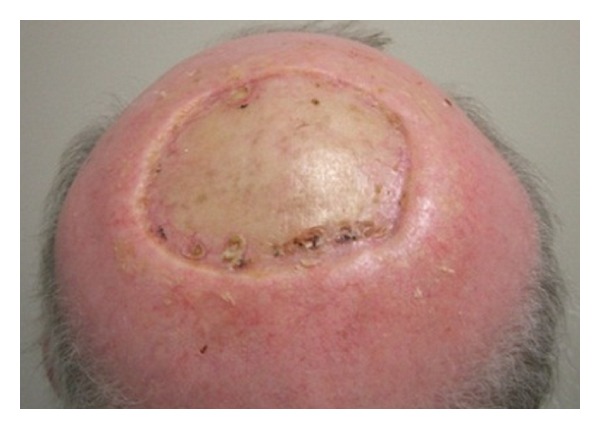
Patient of Case 1 is disease-free at a postoperative followup of 4 months (posterior view of the scalp). At three weeks after the completion of all radiotherapy, the tumour was treated with surgical excision and split-thickness skin graft.

RT was delivered via external beam radiotherapy (EBRT) in a split course with a rest period of fourteen days in between to allow tumour shrinkage and haemostasis. For volumes (see Table 1), we defined the gross tumour volume (GTV) as the obvious clinical tumour by direct observation and the clinical target volume (CTV) as the GTV with a margin of two centimetres all around, and a further expansion of one centimetre to planning target volume (PTV). Initially 20 Gray (Gy) in 5 daily fractions was delivered via opposed 6 MV fields to the midplane of the lesion with the dressing in place (Figure 1). Two weeks after the initial RT, the dressing was taken down and complete haemostasis was observed (Figure 2). Volumetric modulated radiotherapy (VMAT) with 40 Gy/20 daily fractions was then delivered to the same radiotherapy volumes sparing the brain (Figure 3). The tumour continued to shrink and had a maximal diameter of 50 millimetres at three weeks after completing all RT. The patient underwent a wide local excision with skin graft (Figure 4). At one-year followup after the completion of RT and surgery, the patient had no sign of recurrence.

**Case 2**

In March 2007, a 60-year-old man was referred with symptomatic gastrointestinal metastases from primary cutaneous melanoma excised from his upper back over a decade ago. In January 2007, the patient was readmitted for the investigation and management of severe symptomatic anaemia. After negative fibre optic endoscopy, capsule endoscopy identified a single melanoma metastasis in the proximal jejunum, just beyond the duodenojejunal (DJ) flexure. Exploratory laparotomy identified a large inoperable mass involving the DJ flexure.

**Figure 5:**
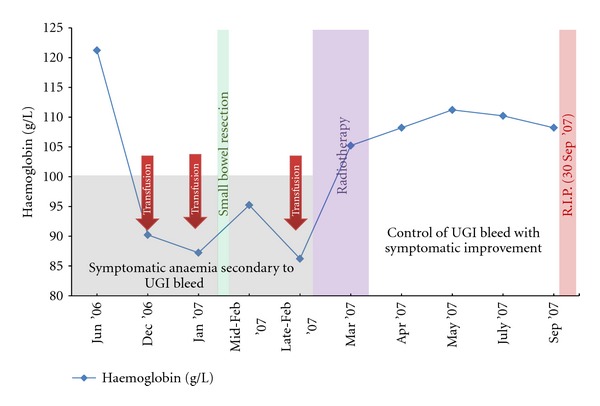
Pre- and postradiotherapy haemoglobin levels of the patient in Case 2. The patient became symptomatic from an occult bleeding caused by a duodenal metastasis of cutaneous melanoma and required continuous transfusion. Palliative radiotherapy of 20 Gy/5 fractions was delivered to control the bleeding. Haemoglobin response to radiotherapy can be appreciated in this graph. (UGI = upper gastrointestinal).

Despite regular transfusional support, his haemoglobin remained symptomatically low. RT was planned with GTV being the lesion as seen on the planning CT with help from coregistration with PET-CT. CTV was made equal to GTV and then expanded by one centimetre to PTV. The mass was irradiated with 20 Gy in 5 daily fractions via opposing anteroposterior/posteroanterior (AP/PA) fields dosed to the midplane. He was treated fasting to decrease the volume of stomach in field. RT was welltolerated. The bleeding ceased and his haemoglobin stabilised with the resolution of his symptoms (Figure 5). He did not require further transfusion.He passed away seven months later due to progression of intracranial disease.

## 3. Discussion

There are a number of modalities conventionally used to manage haemorrhage in patients with advanced malignancy, as reviewed by Pereira and Phan [1]. Systemic treatments include blood products, vitamin K, vasopressin or desmopression, somatostatic analogues, and antifibrinolytic agents. Local modalities include topical haemostatic agents, dressings, endoscopy, vessel ligation, tissue resection, cauterisation, styptics, transcutaneous arterial embolization, or balloon placement. The use of RT is also increasing, as demonstrated by numerous case reports and series [3, 4, 6–9]. However, comparative and controlled trials are still lacking in the role of RT as a local haemostatic agent in malignancies.

The exact mechanism underlying the ability of RT to control malignant bleeding is unclear. RT might work by damaging malignant blood vessels, which are more abundant in haemorrhagic tumours, such as renal cell carcinoma and melanoma. Radiation may affect the pathophysiological process of malignant vessels through inducing malignant endothelial cell damage secondary to extensive DNA damage. It may also cause the activation of signal transduction pathways that can lead to cell cycle arrest or apoptosis [10]. Bleeding in tumours can also be secondary to vascular injury from local tumour invasion, or a systemic coagulopathy caused by paraneoplastic syndromes [1, 11]. However, RT will not stop bleeding from normal vessels that have been invaded by cancerous cells, for example, carotid blowouts in progressing head and neck malignancy. Future studies in this area are warranted.

RT has been used for the control of bleeding in many types of cancer. Langendijk et al. demonstrated in a small series of patients with nonsmall cell lung cancer that EBRT was effective in reducing haemoptysis in 83% [2]. Haemostatic RT has been utilised in haemorrhagic carcinoma of the uterine cervix, with the bleeding control rate of 100% 12–48 hours after EBRT [7]. RT has also been shown to be highly effective in achieving haemostasis in unresectable gastric cancers [3, 4, 8]. A retrospective review of 30 patients with gastric cancer bleeding who underwent palliative RT found improvement in haemoglobin levels and decreased need for blood transfusions in 91% of cases [8]. In a recent retrospective study of 62 patients with advanced cancer, the role of haemostatic RT was evaluated in reducing malignant bleeding [5]. Cihoric et al. found a significant bleeding response, with control rates of 95% for uterovaginal lesions, 100% for the lung lesions, and 90% for upper GI lesions at the end of RT. To date, this study is one of the largest retrospective studies evaluating the role of haemostatic RT in various different types of malignancies; however, skin malignancies were not been included in this study.

A number of fractionation regimens for control of bleeding in malignancies have been employed, and all seem to be effective. In patients with gastric cancer bleeding, dose fractionation regimen has ranged from an 8-Gy single fraction to 40 Gy in 16 fractions [3, 4, 8]. Asakura et al. found that a schedule of 30 Gy in 10 fractions using a 6-, 10-, or 18-MV linear accelerator was sufficient for palliation of bleeding caused by advanced gastric cancer [9]. Langendijk and colleagues treated haemoptysis in lung cancer patients with initial 45 Gy in 20 fractions using opposing AP/PA fields, followed by an additional 15 Gy in 6 fractions to the boost target volume [2]. In a review by Periera and Phan, total doses of 20 Gy via multiple fractions or 8 Gy in a single fraction have been used with success in patients with haemoptysis caused by lung cancer [1]. Barnes and colleagues used 24 Gy in three fractions, delivered on days 0, 7, and 21 over 3 weeks, as their palliative RT regimen for patients with nonmelanoma skin cancers [6]. The “0-7-21” palliative RT showed impressive outcomes, with symptom palliation in 61.3% of their followup patients. However, this retrospective study was compounded by various factors including a small sample size, short followup periods for assessment of late effects, and elderly subjects who were likely to die from other causes [6]. In both our cases, we used a total dose of 20 Gy as a standard approach for both our cases, and this dose was adequate for haemostasis.

Our two cases demonstrate that radiation can also cause haemostasis in bleeding skin malignancies in neoadjuvant and palliative settings. In both of these cases, malignant bleeding presented a life-threatening event and was contained successfully by RT. From our report, we suggest that the optimal RT total dose and fractionation for attaining haemostasis in skin cancer is 20 Gy in 5 fractions. This controlled malignant bleeding even in large tumours. Future studies may consider this dose. To our knowledge, this is the first report demonstrating the effectiveness of RT in the control of bleeding skin malignancies. The publication of the fact that RT can control this type of bleeding may be of benefit to the skin cancer community.

## Supplementary Material

Supplementary Figure 1. Anterior view of the lesion during the second course of radiotherapy (40 Gy/20 fractions) in the patient of Case 1. Image A is taken at 34 Gy (that is, 14 Gy after the initial 20 Gy) and image B is taken at 40 Gy (that is, 20 Gy after the initial 20 Gy). Reduction in tumour size can be appreciated here.

## Figures and Tables

**Table 1:** Definition of volumes in radiotherapy.

Gross tumour volume (GTV)	The gross, palpable, or visible/clinically demonstrable location and extent of the malignant growth

Clinical target volume (CTV)	Anatomical concept-defines a tissue volume that contains a GTV and/or subclinical microscopic malignant disease, which has to be eliminated.

Planning target volume (PTV)	Geometrical concept-selects appropriate beam sizes and arrangements, while taking into account all the possible geometrical variations and inaccuracies.
