# Supplementary material for: Radiotherapy Can Cause Haemostasis in Bleeding Skin Malignancies
Source: Case Rep Med. 2012 Dec 30;2012:168681. doi: 10.1155/2012/168681 (PMC3546482; doi:10.1155/2012/168681)
Supplement: Supplementary file 1 — Supplementary Figure 1. Anterior view of the lesion during the second course of radiotherapy (40 Gy/20 fractions) in the patient of Case 1. Image A is taken at 34 Gy (that is, 14 Gy after the initial 20 Gy) and image B is taken at 40 Gy (that is, 20 Gy after the initial 20 Gy). Reduction in tumour size can be appreciated here. [file 168681.f1.pdf]

SUPPLEMENTARY MATERIAL

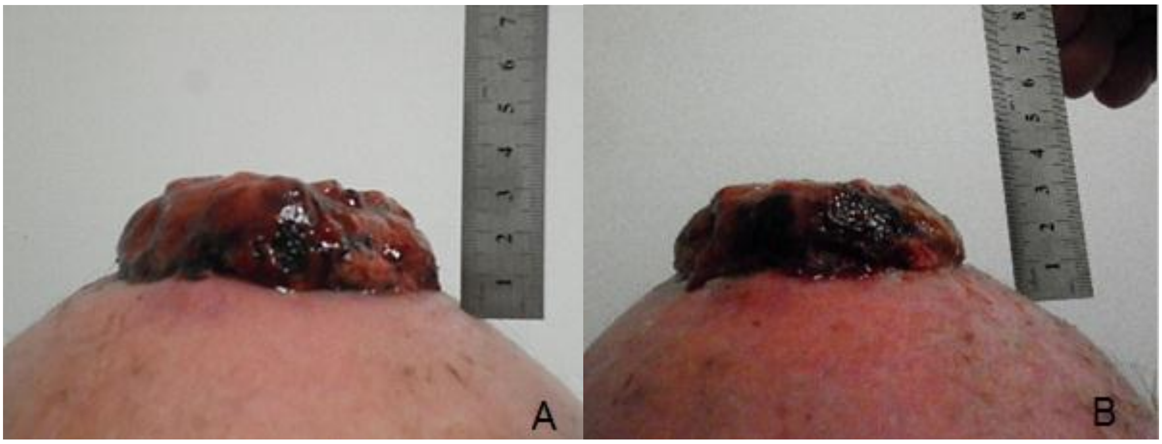

Supplementary Figure 1. Anterior view of the lesion during the second course of radiotherapy (40 Gy/20 fractions) in the patient of Case 1. Image A is taken at 34 Gy (that is, 14 Gy after the initial 20 Gy) and image B is taken at 40 Gy (that is, 20 Gy after the initial 20 Gy). Reduction in tumour size can be appreciated here.
